# Case Report: Asymptomatic SARS-COV2 infection triggering recurrent Takotsubo syndrome

**DOI:** 10.3389/fcvm.2024.1418316

**Published:** 2024-05-24

**Authors:** Gianni Dall’Ara, Miriam Compagnone, Roberto Carletti, Sara Piciucchi, Elisa Gardini, Marcello Galvani

**Affiliations:** ^1^ Department of Medical and Surgical Sciences (DIMEC), University of Bologna, Forlì Campus, Forlì, Italy; ^2^ Cardiology Unit, Morgagni-Pierantoni Hospital, Forlì, Italy; ^3^ Department of Radiology, Morgagni-Pierantoni Hospital, Forlì, Italy; ^4^ Cardiovascular Research Unit, Myriam Zito Sacco Heart Foundation, Forlì, Italy

**Keywords:** Takotsubo, stress cardiomyopathy, apical ballooning syndrome, recurrence, COVID, SARS-CoV-2

## Abstract

Takotsubo syndrome (TTS) is a rare disease mimicking acute coronary syndrome, often triggered by physical or emotional stress, and characterized by transient left ventricular dysfunction. Recurrences are described in about 5% of cases and may have different clinical and imaging patterns. In the present report, SARS-COV-2 infection, even in the absence of symptoms and overt emotional stress, seems correlated with recurrence of TTS, due to the absence of other recognized triggers. The hypothesis is that in predisposed patients, events like catecholamine-induced myocyte injury, direct viral damage, cytokine storm, immune-mediated damage, and procoagulant state, all possibly induced by the infection, may elicit endothelial dysfunction as substrate for TTS onset.

## Introduction

The coronavirus disease-2019 (COVID-19) is mainly a respiratory syndrome with variable severity, but with documented evidence of potential cardiovascular involvement, such as myocarditis, pericarditis, thromboembolism, arrhythmias, Kawasaki's disease, acute coronary syndrome (ACS), and Takotsubo syndrome (TTS). Patients hospitalized for COVID-19 complicated by myocardial injury, identified as an increase in markers of myocardial cell necrosis, have a higher risk of in-hospital mortality (1). TTS, also known as stress cardiomyopathy, is a rare syndrome with well-known clinical manifestations most of the times mimicking, in its early phase, an ACS. Indeed, chest discomfort, ST-T changes on electrocardiogram (ECG), and plasmatic troponin elevation are often reported. TTS is responsible for 1%–2% of hospital admissions with suspected ACS. It is typically characterized by transient left ventricular dysfunction not associated with coronary artery obstruction or extending beyond a coronary distribution. A physical or emotional stress, often part of the medical history, is known to trigger the disease. However, there are few certainties about the pathophysiological mechanisms underlying the syndrome (2).

## Case description

**Figure 1:**
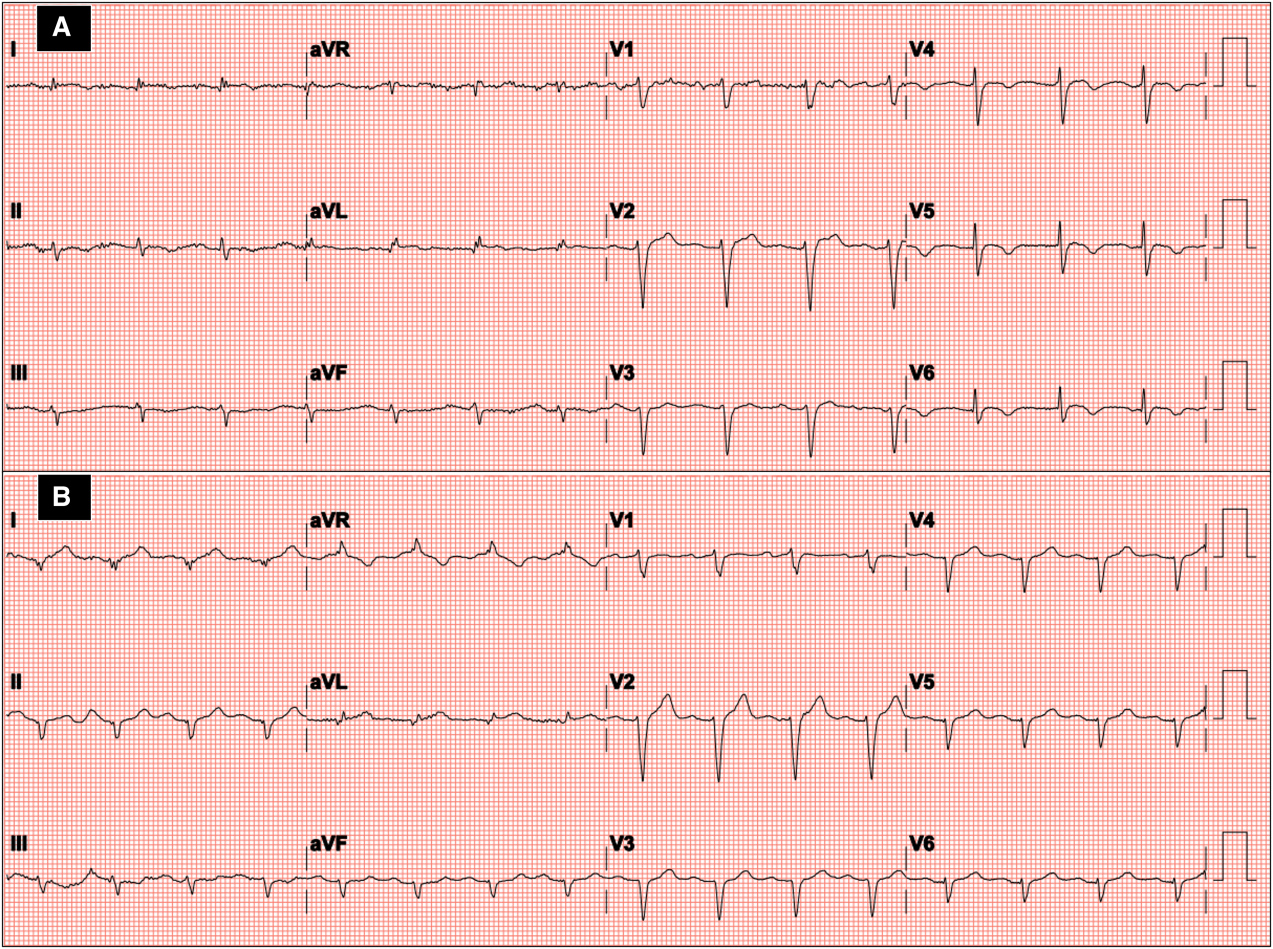
Twelve-lead ECG at hospital admission in the first episode (**A**) and recurrence of Takotsubo syndrome (**B**).

**Figure 2:**
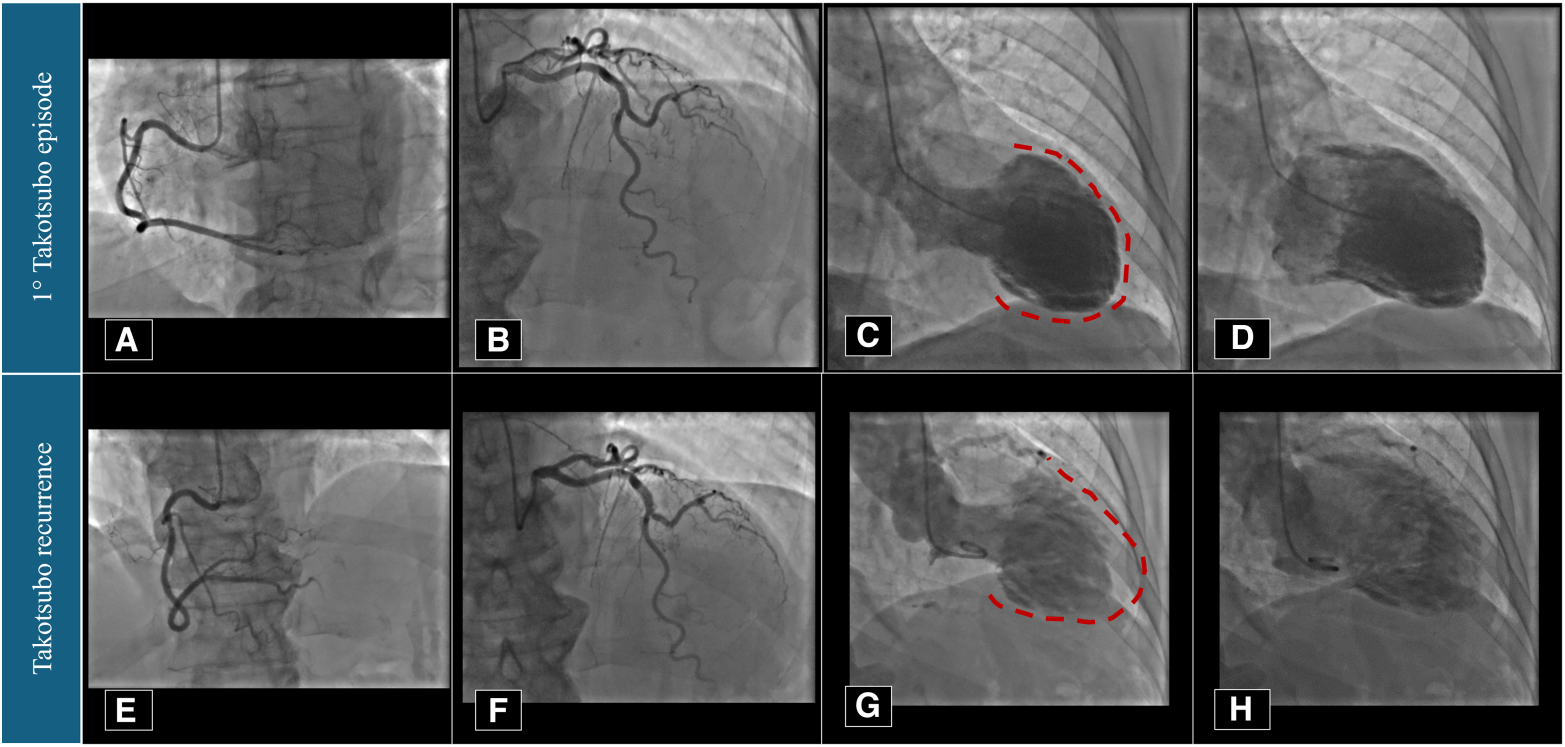
Coronary angiography and left ventriculography imaging performed in 2021 (**A**–**D**) and 2023 (**E**–**H**). Red dotted lines underline the apical akinesia during left ventricular systole.

**Figure 3:**
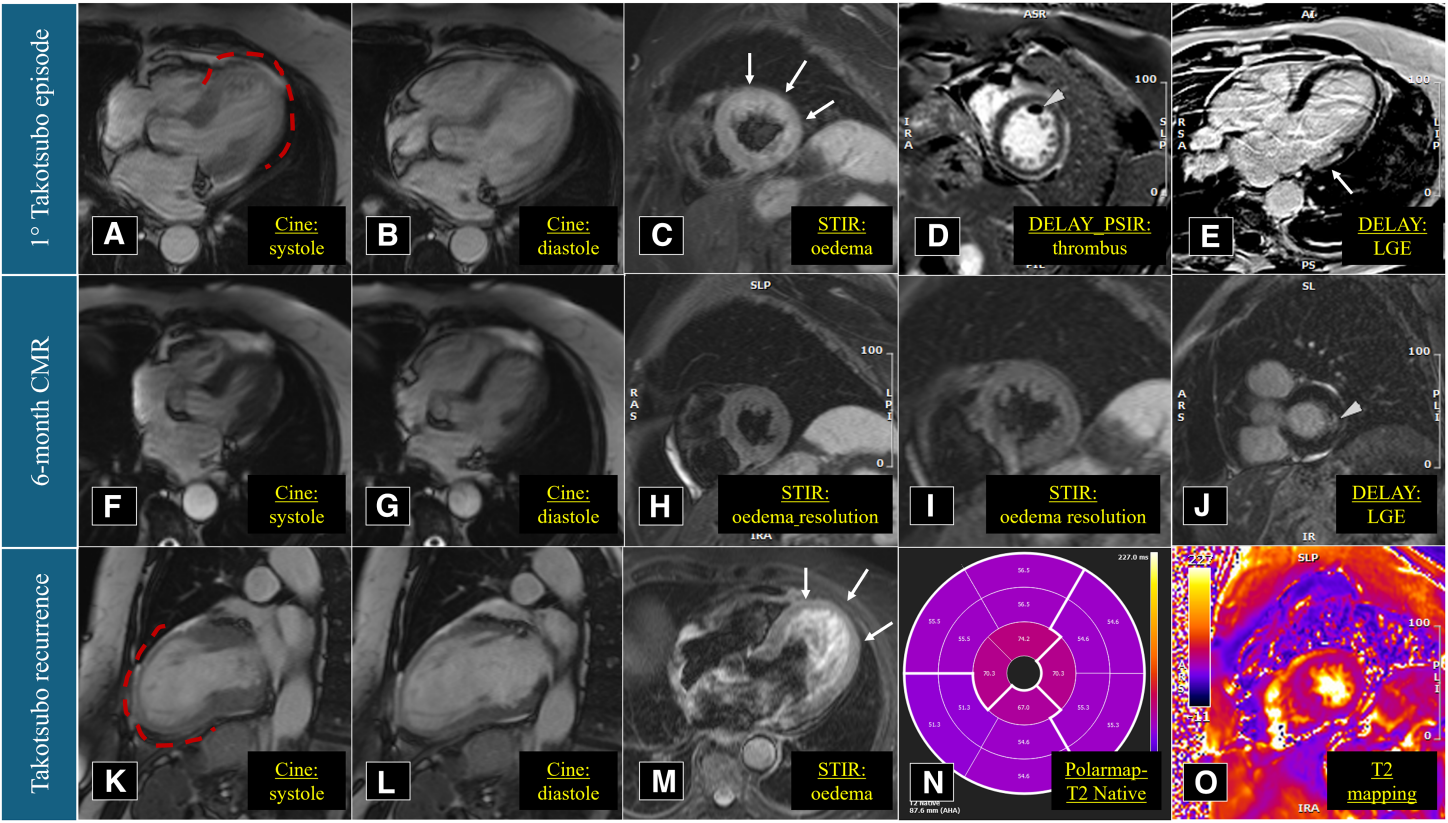
Cardiac magnetic resonance imaging performed in March 2021 (**A**–**E**), September 2021 (**F**–**J**), and March 2023 (**K**–**O**). Red dotted lines underline the apical akinesia during left ventricular systole.

The patient is a 74-year-old woman with type 2 diabetes mellitus, hypercholesterolaemia, mild overweight (25.7 Kg/m^2^), and a remote undocumented history of myocardial infarction in the infero-lateral wall treated by intravenous fibrinolysis. Back then, no coronary artery disease was found at angiography. More recently, in 2021, she was admitted to hospital with a diagnosis of TTS. In that case, she complained of new onset chest pain following an argument on the phone. The 12-lead ECG showed negative T-waves in the infero-lateral leads (Figure 1A). The coronary angiography documented tortuous epicardial coronary arteries, free from significant lesions, with diffuse slow flow (Figures 2A,B). Hyperkinesia of the basal segments, akinesia of the middle and apical segments, and ejection fraction (EF) of 35% were seen at left ventriculography (Figures 2C,D). White blood cell count was 6,690/mmc (URL 10,000/mmc), peak plasma level of high-sensitive troponin-T was 216 ng/L (female gender URL <10), whereas urinary metanephrines were within normal limits. On day 5, a cardiac magnetic resonance (CMR) confirmed the diagnosis (Figures 3A–E) showing in addition the presence of a small intraventricular thrombotic formation (15 × 8 mm) for which oral anticoagulant therapy was given. Discharge therapy included an angiotensin-converting enzyme (ACE) inhibitor. Oral beta blocker was not prescribed in consideration of the occurrence of significant bradycardia on that drug. The clinical course was uncomplicated and, 6 months after the event, a planned CMR found complete functional recovery of the left ventricle, oedema reduction, disappearance of the intraventricular thrombus, subendocardial late gadolinium-enhancement (LGE) at the basal inferolateral wall level (Figures 3F–J) as a result of remote necrosis. Oral anticoagulation was stopped.

In early 2023, the patient arrived at the Emergency Room complaining of fatigue and chest pain. Since the ECG showed slight QRS widening, left anterior hemiblock, and ST-segment elevation in the lateral leads (Figure 1B), the patient underwent an immediate invasive angiography, which excluded again the presence of coronary artery stenosis and showed the apical ballooning pattern of the left ventricle, as in the previous episode (Figures 2E–H). The diagnosis was recurrent TTS. Research of obvious physical or emotional stress was negative. The only relevant finding was a positive routine swab test for severe acute respiratory syndrome coronavirus-2 (SARS-COV-2), in the absence of fever or respiratory symptoms. The hospital stay was prolonged due to transient QT-segment elongation, but no complications occurred. In consideration of the paucity of data about TTS relapse, the patient performed a third CMR. The imaging pattern was similar to that observed 2 years before: extended akinesia at the level of the middle and apical segments, which appeared diffusely oedematous in the STIR sequences; stable subendocardial LGE with ischemic pattern at the basal infero-lateral wall (Figures 3K–O). We repeated the urinary metanephrine assay which demonstrated a slight, but not significant, increase in normetanephrine. WBC count was 5,450/mmc (URL 10,000/mmc), plasmatic interleukin-6 title was 44.7 pg/ml (URL < 5.9), while high-sensitive troponin-T peak was 386 ng/L (female gender URL <10).

Low dose of bisoprolol was added to therapy before discharge. At 1-month follow-up, a trans-thoracic echocardiography showed the left ventricle with normal size, complete recovery of the systolic function (EF 64%), mild diastolic dysfunction. The patient had no further clinical events at 1-year.

## Discussion

TTS is one of the possible cardiovascular complications of COVID-19, characterized by myocardial injury, left ventricular dysfunction, and clinical features of heart failure or ACS. In these patients, TTS may typically manifest as chest pain (38%) associated with respiratory symptoms, but in few cases with cardiac symptoms only (7.7%). Cardiac troponins are often elevated (>80%) and, notably, in-hospital mortality is high (18%–19%) (3, 4).

We reported a case of TTS recurrence in a 74-year-old lady, without a clear physical or emotional stress, but silent SARS-COV-2 infection with elevated inflammation markers. Noteworthy, recurrence is a rare circumstance for this uncommon disease. Singh et al. reported an annual recurrence rate of approximately 1.5%. It is estimated that 2%–5% of patients discharged to home may be affected by a second or more episodes of TTS in a 6-year period (5). Lau et al. observed a median time to recurrence of 2.87 years (6). In our patient, in line with previous data, in spite of differences in term of presence of a stressor, clinical presentation, and ECG alterations, the second episode presented with the same angiographic and CMR pattern (Table 1) (7). This can raise the hypothesis that the underlying myocardial injury may be similar despite possible different triggers. Indeed, the incidence of TTS during the COVID-19 pandemic increased likely due to the intense emotional stress caused by fear of getting seriously ill, fear for loved ones, changes to lifestyle habits including unemployment, economic crisis, and social distancing (8). On the other hand, in COVID-19 patients, irrespective of the severity of the clinical manifestation, several conditions which may favour TTS onset can occur: like catecholamine-induced myocyte injury and neurogenic myocardial stunning, cytokine storm, immune-mediated damage, direct viral myocyte injury, procoagulant state, microvascular coronary impairment due to endothelial injury, and vasospasm (9, 10). The hypothesis is that SARS-COV-2 infection, even asymptomatic, may precipitate TTS onset in predisposed patients, where endothelial dysfunction plays a pivotal role as cause of microvascular impairment (11). The existence of an individual susceptibility can likely manifest itself with episodes of relapse many years apart, in a different social and emotional context, with a different trigger event or in its absence (12). Interestingly, cases of TTS associated with Influenza A and B infection are reported (13).

**Table 1:** Timeline.

Sequence of events	1st episode of Takotsubo syndrome	Recurrence of Takotsubo syndrome
Presentation	Chest pain	Fatigue and chest pain
ECG	Negative T-waves in infero-lateral leads	ST-segment elevation in the lateral leads
Possible trigger	Argument on the phone	Asymptomatic SARS-COV-2 infection
Angiography	• No coronary stenosis, but slow flow • Left ventricle apical ballooning	• No coronary stenosis, but slow flow • Left ventricle apical ballooning
CMR	Hypokinesia of the mid-apical segments, with myocardial oedema	Akinesia of the mid-apical segments, with myocardial oedema
Outcome	Complete recovery	Complete recovery

TTS incidence is higher in postmenopausal women and its recurrence is similarly observed more frequently in the female gender. Conversely, several studies report a slightly higher incidence of SARS-COV-2 infection related TTS cases in males (14). Our patient was not taking a beta-blocker at the time of TTS recurrence. Some observational data found a protective effect of this therapy, but controversy exist about the efficacy of both beta-blockers and ACE-inhibitors administration (6, 15, 16). In consideration of the possible TTS onset without clinical suspicion of COVID-19 disease, its incidence in this setting may be underestimated. Therefore, the diagnosis can be missed or misdiagnosed as ACS or viral myocarditis (17).

## Conclusion

SARS-COV-2 infection, irrespective of symptoms, is correlated with a higher risk of TTS and its recurrence.

## Data Availability

The raw data supporting the conclusions of this article will be made available by the authors, without undue reservation.
